# Monitoring Ras Interactions with the Nucleotide Exchange Factor Son of Sevenless (Sos) Using Site-specific NMR Reporter Signals and Intrinsic Fluorescence*

**DOI:** 10.1074/jbc.M115.691238

**Published:** 2015-11-12

**Authors:** Uybach Vo, Navratna Vajpai, Liz Flavell, Romel Bobby, Alexander L. Breeze, Kevin J. Embrey, Alexander P. Golovanov

**Affiliations:** From the ‡ Manchester Institute of Biotechnology and Faculty of Life Sciences, The University of Manchester, 131 Princess Street, Manchester M1 7DN, United Kingdom and; § Discovery Sciences, AstraZeneca, Mereside, Alderley Park, Cheshire SK10 4TF, United Kingdom

**Keywords:** allosteric regulation, nuclear magnetic resonance (NMR), protein-protein interaction, Ras protein, small GTPase, Sos protein

## Abstract

The activity of Ras is controlled by the interconversion between GTP- and GDP-bound forms partly regulated by the binding of the guanine nucleotide exchange factor Son of Sevenless (Sos). The details of Sos binding, leading to nucleotide exchange and subsequent dissociation of the complex, are not completely understood. Here, we used uniformly ^15^N-labeled Ras as well as [^13^C]methyl-Met,Ile-labeled Sos for observing site-specific details of Ras-Sos interactions in solution. Binding of various forms of Ras (loaded with GDP and mimics of GTP or nucleotide-free) at the allosteric and catalytic sites of Sos was comprehensively characterized by monitoring signal perturbations in the NMR spectra. The overall affinity of binding between these protein variants as well as their selected functional mutants was also investigated using intrinsic fluorescence. The data support a positive feedback activation of Sos by Ras·GTP with Ras·GTP binding as a substrate for the catalytic site of activated Sos more weakly than Ras·GDP, suggesting that Sos should actively promote unidirectional GDP → GTP exchange on Ras in preference of passive homonucleotide exchange. Ras·GDP weakly binds to the catalytic but not to the allosteric site of Sos. This confirms that Ras·GDP cannot properly activate Sos at the allosteric site. The novel site-specific assay described may be useful for design of drugs aimed at perturbing Ras-Sos interactions.

## Introduction

Ras proteins are mutated in 30% of all human tumors, contributing to several malignant phenotypes, including abnormal cell growth, proliferation, and apoptosis (1). The activity of Ras is controlled by the interconversion between GTP- and GDP-bound forms with GTP binding required for the active form (2). Ras activation is mediated through the binding of guanine nucleotide exchange factors of which the most important is Son of Sevenless (Sos),^5^ which stimulates the release of bound GDP from Ras and is currently thought to facilitate the more abundant cytosolic GTP to bind in its place (3). The major Ras isoforms, H-Ras, K-Ras, and N-Ras, are closely related, bearing 85% amino acid sequence identity; however, 85% of clinically observed Ras mutations occur in K-Ras (4). Point mutations at codon 12 impair the GTPase activity of Ras isoforms both by preventing productive binding of GTPase-activating proteins that accelerate GTP hydrolysis and by suppressing intrinsic (basal) GTP hydrolase activity. The single point mutation G12V in K-Ras, which is a common variant found in human tumors, causes constitutive activation of Ras (5). This leads to an accumulation of the GTP-bound active form of K-Ras (6).

Over the last few decades, studies have shown that several regions of Ras are of particular interest for control of its functional cycle and present potential intervention sites for new therapeutics (4–7). The P-loop (residues G10–S17) is responsible for phosphate binding, whereas the Switch I (Y32–Y40) and Switch II (G60–T75) regions are critical for interactions with guanine nucleotide exchange factors (7) and effector proteins (8). Sos contains two domains that are essential for Ras nucleotide exchange, namely the Ras exchanger motif domain and the Cdc25 domain. The latter contains the catalytic site in which bound Ras undergoes nucleotide exchange (9, 10). A second Ras molecule binds at a distal (also called allosteric) site, which is located between the Ras exchanger motif and Cdc25 domains. Binding of Ras·GTP to the allosteric site induces a conformational change that is propagated to the catalytic site (10–12), enhancing the rate of Ras nucleotide exchange activity by increasing the affinity of Ras at the catalytic site. However, it remains unclear whether or to what extent GDP-loaded Ras can allosterically activate Sos (11–13) and whether different forms of Ras bind at the catalytic site of activated Sos equally well. One current model is that weak binding of Ras·GDP at the distal (allosteric) site causes basal activation of Sos for further catalytic site binding (11) with stronger Ras·GTP binding at the distal site promoting a significantly higher level of activation, resulting in a positive feedback activation mechanism (10–12). Additionally, the binding at the allosteric site (and hence the activation of Sos) is regulated and autoinhibited by interactions with neighboring histone, Dbl homology (DH), and pleckstrin homology (PH) domains of Sos (11, 14).

Currently, nucleotide exchange at the catalytic site is presumed to be passive: GDP is exchanged for GTP driven through the higher cytosolic concentration of GTP (3). For convenience, currently used nucleotide exchange assays often quantify the homonucleotide exchange, GDP → GDP or GTP → GTP, as a measure of biological activity of Sos (10, 11, 13). However, it is unclear whether or how rebinding of GDP or GTP to nucleotide-free Ras (a presumed transition state) at the catalytic site of Sos occurs and how this triggers the dissociation of Ras upon completion of nucleotide exchange, allowing Sos to engage in further cycles of guanine nucleotide exchange factor activity. Binding of Ras·GTP at the allosteric site and thus activation of Sos increases affinity of Ras·GDP binding at the catalytic site with a measured *K_d_* of 1.9 μm (11), but to our knowledge, the affinity of Ras·GTP binding to the catalytic site of activated Sos has not been reported to date.

The model of Sos activation by binding of Ras to the allosteric site is based on static snap shots from x-ray crystal structures (10, 11) and therefore may not fully represent the dynamic subtleties of the process in solution. Indeed, recent single molecule studies have suggested a critical role for dynamic fluctuations in the allosteric activation of Sos by Ras·GTP and hinted at the possibility that Ras·GDP can also activate Sos (13). How these fluctuations are modulated at a detailed structural level and whether Ras·GDP can bind with sufficient affinity to the allosteric site to activate Sos remain to be established.

Previous solution NMR studies, which can capture the dynamic behavior of proteins, have explored the process of GTP hydrolysis and nucleotide exchange in H-Ras (15–17). Other studies were able to demonstrate that H-Ras interacts with its downstream effector proteins such as Raf kinases (18–20). Studies using ^31^P NMR spectroscopy revealed that the GTP-bound form of Ras is likely to exist in two or more conformational states that interconvert on the millisecond time scale (21). The conformational equilibrium can be shifted by introducing point mutations that enhance the affinity of GTP-Ras for Raf kinases (21–25).

To our knowledge, no detailed solution studies of the Ras-Sos interactions using NMR spectroscopy have yet been reported. Here, we used NMR to dissect Ras-Sos interactions. Signal perturbations were used to monitor changes in Ras and Sos upon binding, depending on their stoichiometry and type of nucleotide present. Moreover, we introduced a number of non-perturbing probes (^13^C-labeled methyls of methionines (26)) into Sos and used them to monitor Ras binding separately to the allosteric and catalytic sites. Using intrinsic fluorescence, we also measured the binding affinity to Sos of wild-type (WT) as well as functional mutants of Ras in various nucleotide-loaded forms. Our data have enabled us to disentangle the binding preferences of GTP- and GDP-loaded forms of Ras in solution at the specific sites on Sos.

## Experimental Procedures

### 

#### 

##### Protein Expression and Purification

H-Ras (residues 1-166), K-Ras (residues 1–166), Sos^Cat^ (residues 563–1049), and Sos^HD-DH-PH-Cat^ (residues 1–1049) gene sequences were synthesized by Geneart (Life Technologies) and cloned into a pET28b vector with an N-terminal His_6_ tag followed by a tobacco etch virus protease cleavage site prior to the protein sequence. Proteins were expressed in BL21-GOLD(DE3) competent cells, and the seeder cultures were grown in Luria broth (LB) medium. All samples were grown using a similar protocol (27) for unlabeled and uniformly ^15^N-labeled H-Ras samples. WT and mutant Sos constructs (Sos^Cat^ and Sos^HD-DH-PH-Cat^) were grown in minimal M9 medium containing 50 μg/ml kanamycin and 12.5 μg/ml tetracycline antibiotics supplemented with micronutrients and vitamins. Cells were then induced by 0.1 mm isopropyl β-d-1-thiogalactopyranoside followed by the addition of 2 g/liter d-glucose. Uniformly recombinant Sos^Cat^ ^13^C-labeled at the Met (ϵ-) and Ile (δ_1_-)methyl positions ([^13^C-methyl-Met,Ile]Sos^Cat^) was produced under a similar protocol in D_2_O but with an additional feed of 200 mg/liter [^13^C]methyl-Met, 120 mg/liter [^13^C]methyl-α-ketobutyrate, and 2 g/liter *d*_7_-d-glucose administered at the point of isopropyl β-d-1-thiogalactopyranoside induction. Protein purification was carried out as described previously (27). Unless stated otherwise, all proteins were cleaved from their N-terminal His_6_ tags using tobacco etch virus protease.

##### Nucleotide Exchange in Ras Samples

Purified H-Ras samples were incubated with a 20-fold excess of GTPγS, GppNp, or GppCp and 1100-fold His-tagged Sos in 50 mm Hepes, 50 mm NaCl, 2 mm MgCl_2_, 2 mm tris(2-carboxyethyl)phosphine, 0.1 mm EDTA, 0.02% NaN_3_ at pH 7.4. The samples were left overnight at 4 °C to allow nucleotide exchange. The mixture was then passed through a nickel-nitrilotriacetic acid column equilibrated with 30 mm Na_2_HPO_4_, 1 mm DTT, 2 mm MgCl_2_, 0.1 mm EDTA, 0.02% NaN_3_ at pH 7.0 to remove the His_6_-tagged Sos and free nucleotide. The samples were concentrated using 0.5-ml VivaSpin concentrators (Sartorius; 10,000 molecular weight cutoff). Protein concentration was determined by standard Bradford assays. Nucleotide exchange was confirmed by electrospray mass spectrometry. For the preparation of the nucleotide-free form of Ras (hereafter referred to as H-Ras^NF^), H-Ras·GDP (200 μm) sample was incubated with 20 mm EDTA to strip the Mg^2+^ and nucleotide from Ras. After 2 h, the sample was passed down a pre-equilibrated Nap-5 column (GE Healthcare) to exchange the sample into Hepes buffer, pH 7.4, containing 50 mm l-Arg and l-Glu to improve sample stability for the duration of NMR measurements (28). H-Ras^NF^ was then concentrated down as required using an Amicon Ultra-15 centrifugal filter unit (Millipore) with a 10,000 molecular weight cutoff.

##### NMR Experiments

All NMR spectra were collected at 298 K (unless stated otherwise) on Bruker 600- and 800-MHz (Avance I and III, respectively) spectrometers equipped with 5-mm TCI CryoProbes with *z*-axis gradients using standard experiments and parameters from the Bruker library. Uniformly ^15^N-labeled H-Ras was used at a concentration of 100 μm, and [^13^C-methyl-Met,Ile]Sos^Cat^ was used at 60 μm with concentrations of added non-labeled protein variants as indicated. NMR samples containing proteins or their mixtures were prepared in either phosphate buffer at pH 7.0 or Hepes buffer at pH 7.4 supplemented with 5% D_2_O and placed in a Shigemi tube. All spectra were processed in Topspin 2.1 or 3.1 and analyzed in NMRViewJ (29).

##### Measuring Affinities of Ras-Sos Interactions

Fluorescence binding assays were performed using a luminescence spectrometer (PerkinElmer Life Sciences) with emission and excitation slits set to 3 and 10 nm, respectively. Samples were measured in 1-ml quartz cuvettes with path lengths of 1 and 0.4 cm used for excitation (295 nm) and emission (336 nm), respectively. Data points were taken in quadruplicate with a scan speed of 300 ns/min. WT Sos^Cat^ or Sos^HD-DH-PH-Cat^ samples (10 μm) were incubated with increasing amounts of Ras (1–50 μm) in Hepes buffer. To obtain the dissociation constant of binding (*K_d_*), the quenching of intrinsic fluorescence of the Trp residues in Sos upon addition of the non-fluorescent Ras protein was monitored. The weak contribution of intrinsic fluorescence of Ras as well as the change in fluorescence due to dilution effects were taken into account and compensated for. Fluorescence experiments were repeated three times. Data points were fitted (change in fluorescence *versus* Ras concentration) using non-linear regression to a standard quadratic binding equation using GraFit (30).

## Results

### 

#### 

##### Fluorescence Measurements

**FIGURE 1.**
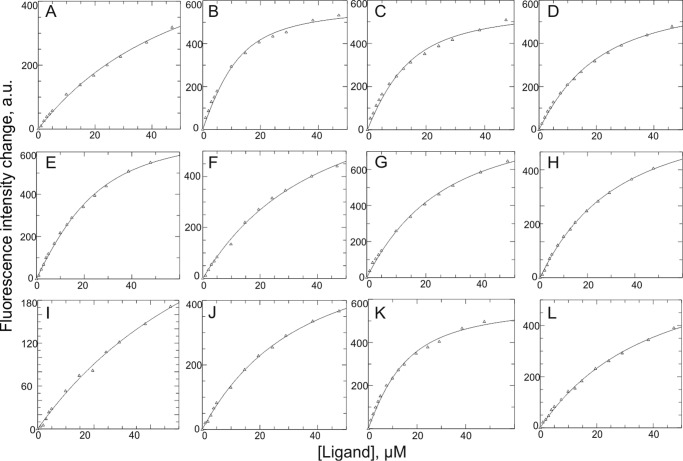
**Change in intrinsic fluorescence signal of Sos^Cat^ variants upon addition of protein ligands, used to determine the dissociation constants *K_d_* presented in Table 1.** Individual panels show binding of WT Sos^Cat^ with H-Ras·GDP (*A*), H-Ras·GTPγS (*B*), H-Ras·GppNp (*C*), H-Ras·GppCp (*D*), or H-RasY64A·GTPγS (*E*) and binding of Sos^Cat^W729E with H-Ras·GTPγS (*F*), Sos^HD-DH-PH-Cat^ with H-Ras·GTPγS (*G*), Sos^Cat^W729E with H-RasY64A·GTPγS (*H*), Sos^Cat^W729E with H-Ras·GDP (*I*), WT Sos^Cat^ (preloaded with 30 μm H-RasY64A·GTPγS) with H-Ras·GTPγS (*J*), WT Sos^Cat^ with K-Ras·GTPγS (*K*), and WT Sos^Cat^ with K-RasG12V·GTPγS (*L*). The fluorescence data were fitted to a single site binding model using GraFit software (30). *a.u.*, arbitrary units.

The overall apparent binding affinities of Ras to Sos were determined by monitoring the change (*i.e.* quenching) in Sos fluorescence upon the formation of the Ras/Sos complex (Fig. 1). This approach relied on the strong intrinsic fluorescence signal of the multiple Trp residues within the Sos^Cat^ and Sos^HD-DH-PH-Cat^. Ras is devoid of Trp residues, and therefore its intrinsic fluorescence signal at 295 nm is negligible. To determine the affinities accurately, the minor dilution effect and the background fluorescence of Ras (without Sos) were further subtracted from the overall fluorescence. First, we explored the differences in binding of the isoforms H-Ras and K-Ras as well as K-RasG12V, a common cancer-associated mutant, to Sos when Ras was loaded with different stable analogs of GTP. Traditionally, nucleotide exchange has been studied using fluorescently labeled analogs such as mantGTP (11, 14, 31); however, the bulky mant adduct has been shown to affect the kinetics of nucleotide exchange and hydrolysis in H-Ras (16, 23). For these reasons, we used unlabeled slowly or non-hydrolyzable GTP mimics (GTPγS, GppNp, and GppCp) in our study coupled with measurement of intrinsic protein fluorescence. Dissociation constants (*K_d_*) for interactions of H-Ras·GTPγS, H-Ras·GppNp, and H-Ras·GppCp with Sos^Cat^ are generally very similar to each other (∼5–8 μm) (Table 1). Conversely, the affinity of H-Ras·GDP for Sos^Cat^ is ∼10-fold weaker (*K_d_* ∼ 54 μm) (Table 1). These results are in agreement with the affinities previously measured for Ras·GDP binding at the allosteric site of non-activated Sos (11). In addition, the binding of K-Ras·GTPγS to Sos^Cat^ (*K_d_* = 10 ± 1 μm) is stronger than the binding of K-RasG12V·GTPγS (*K_d_* = 31 ± 2 μm) but comparable with binding affinities of H-Ras loaded with GTP analogs (Table 1). This suggests that WT K-Ras and H-Ras have fairly similar Sos binding properties, whereas the oncogenic mutation K-RasG12V has reduced binding affinity to Sos.

**TABLE 1:** **Summary of macroscopic dissociation constants for Ras/Sos complexes measured by fluorescence** Protein variants used for each titration experiment as well as a brief description of the expected functional effect of mutation are indicated.

Description of expected effect of mutation used	Sos variant present*^a^*	Ras variant added	Measured *K_d_*
			μ*m*
None	Sos^Cat^	H-Ras·GDP	54 ± 4
None	Sos^Cat^	H-Ras·GTPγS	5.0 ± 0.8
None	Sos^Cat^	H-Ras·GppNp	6.0 ± 0.9
None	Sos^Cat^	H-Ras·GppCp	8.0 ± 1.0
Is not expected to bind at catalytic site	Sos^Cat^	H-RasY64A·GTPγS	10 ± 1
Binding expected to be hindered at allosteric site	Sos^Cat^W729E	H-Ras·GTPγS	28 ± 1
Binding expected to be hindered at allosteric site	Sos^HD-DH-PH-Cat^	H-Ras·GTPγS	24 ± 2
Binding expected to be hindered at both sites of Sos	Sos^Cat^W729E	H-RasY64A·GTPγS	32 ± 3
Binding expected to be hindered at allosteric site	Sos^Cat^W729E	H-Ras·GDP	67 ± 2
H-RasY64A·GTPγS expected to partially saturate allosteric site	Sos^Cat^ (preloaded with 30 μm H-RasY64A·GTPγS)	H-Ras·GTPγS	21 ± 2*^b^*
None	Sos^Cat^	K-Ras·GTPγS	10 ± 1
Oncogenic variant	Sos^Cat^	K-RasG12V·GTPγS	31 ± 2

*^a^* Unless stated otherwise, the WT version of protein was used.

*^b^* Lower limit estimate.

To assess the affinity of Ras binding specifically at the catalytic site of Sos (when Sos is not activated), we set up further experiments so that the allosteric site of Sos was either obstructed, as in the Sos^HD-DH-PH-Cat^ variant, or disrupted by mutation, as in Sos^Cat^W729E (10–12, 14, 32, 33). The affinity of H-Ras·GTPγS for Sos^HD-DH-PH-Cat^ (*K_d_* = 24 μm) and for Sos^Cat^W729E (*K_d_* = 28 μm) is significantly weaker than H-Ras·GTPγS binding to WT Sos^Cat^ (*K_d_* = 5 μm; Table 1). The binding of H-Ras·GDP to Sos^Cat^W729E (*K_d_* = 67 μm) is weaker than the binding of H-Ras·GTP to the same construct (Table 1).

Interestingly, when the allosteric site of Sos^Cat^ is partially saturated by the addition of excess H-RasY64A·GTPγS, a mutant that cannot bind to the catalytic site of Sos (11), the fluorescence measurements reveal that the binding of WT H-Ras·GTPγS at the catalytic site is weak with an observed *K_d_* of 21 μm (Table 1). This value provides an upper limit estimate for the binding affinity to the catalytic site as much of the observed affinity may be due to competition between the Ras forms for the allosteric site. Although the addition of RasY64A·GTPγS in this experiment was expected to activate Sos for further catalytic site binding (11, 12), our results suggest that H-Ras·GTPγS has only a modest-to-weak affinity (*K_d_* ≥ 21 μm) for the catalytic site of Sos. This result complements earlier studies in which loading of H-RasY64A·GppNp at the allosteric site was found to increase the affinity for Ras·GDP at the catalytic site with a *K_d_* of 1.9 μm (11). GppNp is another analog of GTP with functional properties similar to those of GTPγS (see Table 1). The weaker binding observed for Ras·GTP at the catalytic site has not been described previously and is significant as it implies that Ras·GTP-activated Sos preferentially binds Ras·GDP at its catalytic site but not Ras·GTP. This suggests a clear preference of activated Sos for the heteronucleotide exchange reaction GDP → GTP at its catalytic site, which we believe has not been recognized previously.

The binding of mutant H-RasY64A (*K_d_* = 10 μm) to Sos^Cat^ was marginally weaker but comparable with WT H-Ras, suggesting that this mutant and the wild-type protein have similar binding properties when they are loaded with GTPγS, and both of them preferentially bind to the allosteric site. Attempts to fit the quantitative binding data to a two-site model found no significant improvement in the fit over the simpler one-site models and are in agreement with Ras·GTPγS preferentially interacting at only one, namely the allosteric, site. To study the site-specific binding of Ras with Sos further, we monitored these interactions using NMR spectroscopy.

##### Monitoring NMR Signal Perturbations of H-Ras upon Binding to Different Nucleotides and Sos

**FIGURE 2.**
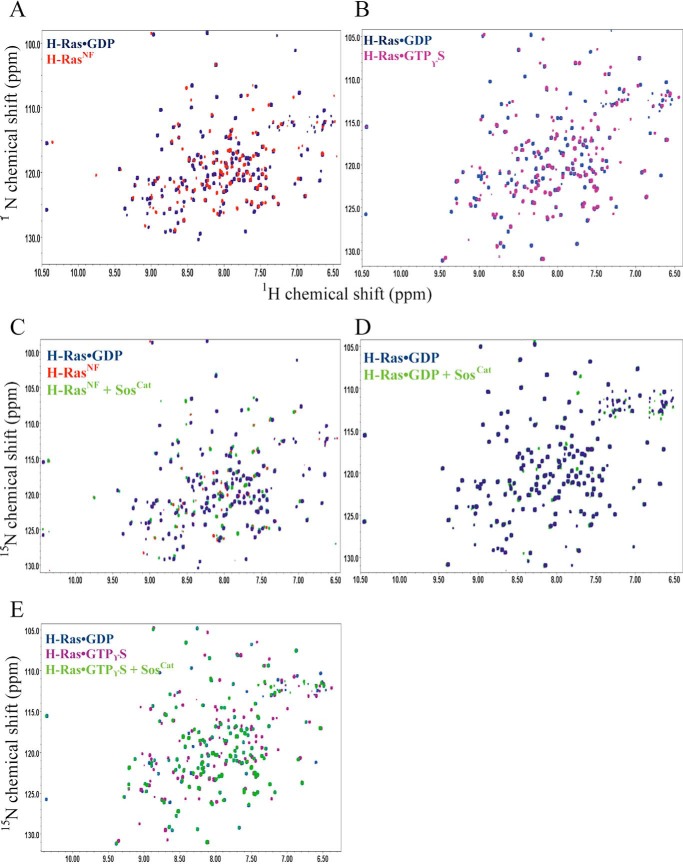
**Differences between H-Ras states in the ^1^H,^15^N TROSY spectra.** *A*, comparison between H-Ras·GDP (*blue*) and H-Ras^NF^ (*red*). *B*, comparison between H-Ras·GDP (*blue*) and H-Ras·GTPγS (*pink*). *C*, spectra of H-Ras bound to GDP (*blue*), H-Ras^NF^ (*red*), and H-Ras^NF^ bound to Sos (*green*) in a 2:1 Ras:Sos stoichiometry. *D*, spectra of H-Ras·GDP (*blue*) and H-Ras·GDP/Sos (*green*) in a 2:1 Ras:Sos stoichiometry. Spectra in *A–D* were collected at 25 °C. *E*, comparison among H-Ras·GDP (*blue*), H-Ras·GTPγS (*magenta*), and H-Ras·GTPγS bound to Sos (*green*) in a 2:1 Ras:Sos stoichiometry at 18 °C.

First, the previous ^1^H^N^ and ^15^N backbone assignments of Ras residues 1–166 (17, 27) were transferred to spectra of nucleotide-loaded and nucleotide-free states to achieve their partial assignment for a number of signals used as reporters. Amide signal perturbations of the GDP- and GTP-bound forms of uniformly ^15^N-labeled H-Ras were then monitored by acquiring ^1^H,^15^N correlation TROSY spectra upon the addition of unlabeled Sos^Cat^ (Fig. 2). The influence of the nucleotide in maintaining the structural integrity of H-Ras was also investigated by comparing the spectra of H-Ras^NF^ with the nucleotide-loaded forms H-Ras·GDP and H-Ras·GTPγS (Fig. 2).

**FIGURE 3.**
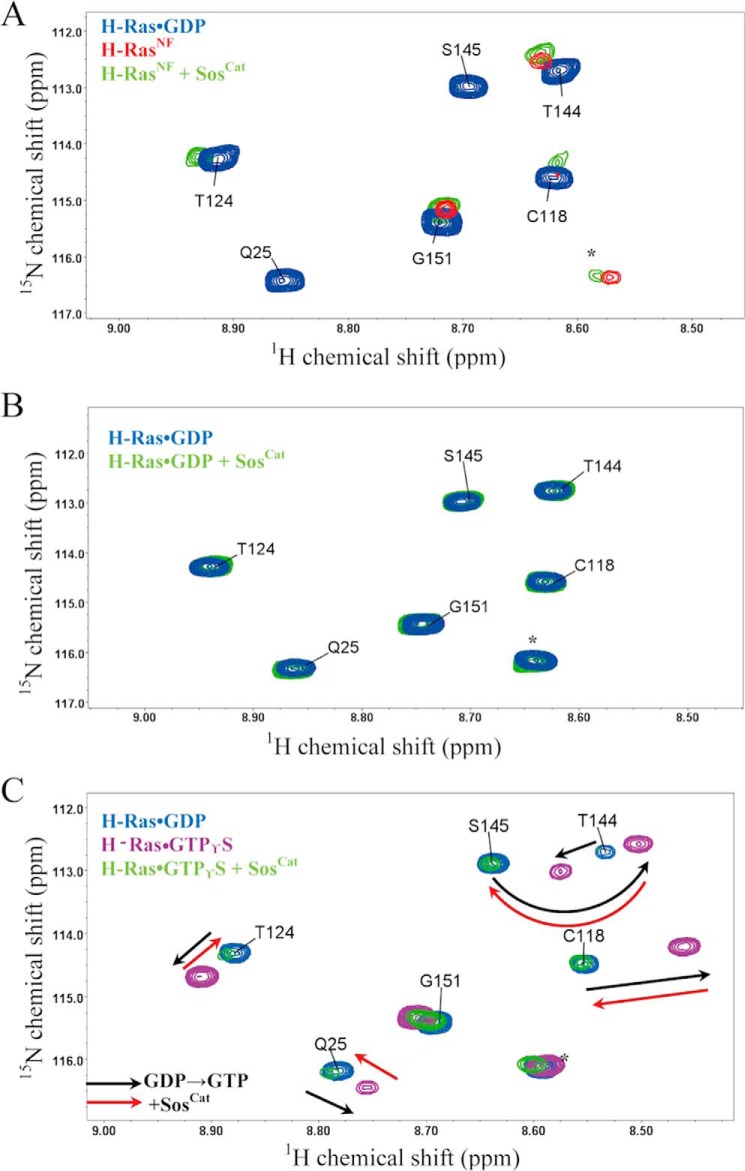
**Overlay of a representative region of the ^1^H,^15^N TROSY spectra of different forms of ^15^N-labeled H-Ras.** *A*, spectra of H-Ras bound to GDP (*blue*), H-Ras^NF^ (*red*), and H-Ras^NF^ bound to Sos (*green*) in a 2:1 stoichiometry recorded at 25 °C. *B*, spectra of H-Ras·GDP (*blue*) and H-Ras·GDP/Sos (*green*) in a 2:1 Ras:Sos stoichiometry recorded at 25 °C. *C*, comparison among H-Ras·GDP (*blue*), H-Ras·GTPγS (*magenta*), and H-Ras·GTPγS bound to Sos (*green*) in a 2:1 stoichiometry recorded at 18 °C. Directions of representative signal shifts due to nucleotide change from GDP to GTPγS are marked with *dashed black arrows*. Directions of the shifts that are caused by the subsequent addition of Sos^Cat^ to H-Ras·GTPγS are marked with *dashed red arrows*. Spectrally aliased side chain resonances are labeled with *asterisks*.

The addition of unlabeled WT Sos^Cat^ to the ^15^N-labeled H-Ras^NF^ sample at a stoichiometry of 2:1 (Ras:Sos) showed clear signal perturbations for a number of Ras residues and the reappearance of two amide peaks belonging to residues C118 and T124 (Fig. 3*A*). These signals, which became somewhat broadened for unbound nucleotide-free Ras^NF^, recovered for Ras^NF^ bound to Sos in positions similar to the GDP-loaded form. The chemical shifts were generally comparable with those caused by GDP binding to H-Ras^NF^. Overall, this result confirms that H-Ras^NF^ and Sos are able to form a complex in solution, and this binding affects Ras dynamics. Our observations are thus consistent with a model whereby binding of Ras at the catalytic site of Sos stabilizes its nucleotide-free conformation and primes it for reloading with GTP (8, 10).

Intriguingly, from our signal perturbation study, it is clear that addition of Sos^Cat^ to ^15^N-labeled H-Ras·GDP does not induce any noticeable changes in its ^1^H,^15^N correlation spectrum with no noticeable effects on peak positions or line widths (Fig. 3*B*), suggesting that this interaction is very weak and much weaker than the interaction with H-Ras^NF^. The *K_d_* of H-Ras·GDP binding with Sos^Cat^ measured by fluorescence is only 54 μm (Table 1). Our solution NMR data corroborate previous findings that basal binding of Ras·GDP with Sos at either site is relatively weak and would require binding of Ras·GTP at the allosteric site to increase the affinity of Sos for Ras·GDP at the catalytic site (10, 11). This Ras·GDP binding contrasts with Ras^NF^, which can bind to Sos directly even in the absence of Ras·GTP (8).

The addition of unlabeled Sos^Cat^ to a ^15^N-labeled H-Ras·GTPγS sample at a 2:1 Ras:Sos stoichiometry revealed some significant changes in the TROSY spectrum (Fig. 3*C*). This provides evidence that H-Ras·GTPγS is able to bind to Sos with higher affinity than H-Ras·GDP and that this increased affinity is accompanied by significant structural and/or dynamic rearrangements for H-Ras. A closer analysis of the signal shift patterns in the NMR spectra of the complexes of Ras with GDP, GTPγS, and GTPγS + Sos revealed that change of GDP for GTP causes significant chemical shift changes for a number of residues such as Q25, C118, T124, and S145, whereas subsequent binding of Sos to Ras·GTPγS shifts its peaks back to positions similar to those in the free Ras·GDP (Fig. 3*C*). This intriguing observation is difficult to explain definitively based on available measurements: because of the presence of two different binding sites for Ras on Sos^Cat^, it is not clear whether these signal changes are characteristic of conformational change upon Ras binding at the allosteric or catalytic sites and/or exchange between the two. Further experiments, *e.g.* using preloading with H-RasY64A·GTPγS, a mutant that can only bind to the allosteric site, or using different ratios of components as well as using ^31^P NMR spectroscopy (21–25), may provide an explanation in the future.

The NMR data presented here clearly show that different forms of Ras, either nucleotide-free or loaded with GDP or GTP, bind Sos with different affinities, causing some characteristic changes in the TROSY spectra of Ras. However, as the P-loop and Switch I and II regions of Ras have been structurally shown to be involved in binding at both catalytic and allosteric sites of Sos (10), it remains challenging to distinguish effects of binding at these two sites when observing the signal perturbations of Ras only. Therefore, to gain better insight into the site-specific interactions of Ras with Sos, we monitored the formation of the same complexes using unlabeled Ras and [^13^C]methyl-Met,Ile-labeled Sos^Cat^.

##### [^13^C]Methyl-Met Resonance Assignment of Sos^Cat^

**FIGURE 4.**
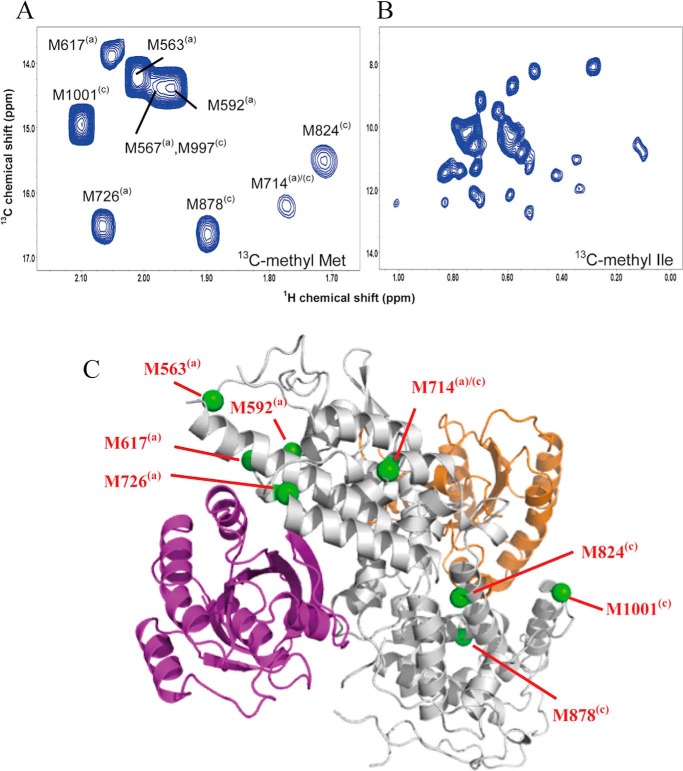
**Site-specific reporter signals of Sos.** Expanded fingerprint regions of the ^1^H,^13^C HMQC spectra of 60 μm Sos show the region with assigned [^13^C]methyl-Met resonances apportioned to allosteric and catalytic sites (*A*) and the region showing non-assigned [^13^C]methyl signals of Ile residues as additional reporter signals (*B*). *C*, position of selected Met residues (highlighted in *green* and labeled individually) in relation to the Ras/Sos crystal structure (Protein Data Bank code 1NVV). Ras molecules bound at the allosteric and catalytic sites of Sos (*gray*) are shown in *purple* and *orange*, respectively. The *superscripts (a)* and *(c)* mark the proximity of Met residues to the allosteric and catalytic sites, respectively.

**FIGURE 5.**
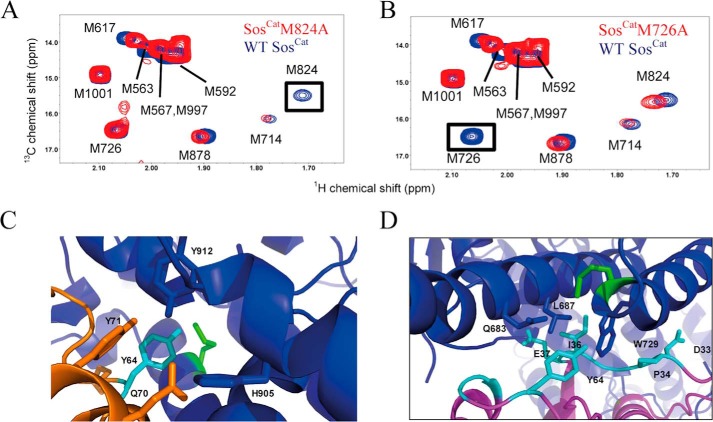
**An example of using mutagenesis to assign methyl signals of Met in Sos^Cat^ and relating Met positions to the structure.** Overlays of [^13^C]methyl region of ^1^H,^13^C HMQC spectra for WT (*blue*) and mutants (*red*) M824A (*A*) and M726A (*B*) of Sos^Cat^. Signals affected by the mutation are enclosed in *boxes*. Structures below show reporter groups of Sos in contact with residues of H-Ras. *C*, structure of the catalytic interface deduced from the crystal structure of H-Ras^NF^ form. H-Ras^NF^ (*orange*) is bound at the catalytic site of Sos (*blue*). Residue M824^(c)^ (*green*) forms hydrophobic interactions with H-Ras Y64 (*cyan*). *D*, M726^(a)^ (highlighted in *green*) interacts with H-Ras at the allosteric site (*purple*). The figures were produced in PyMOL.

To monitor Ras binding at the allosteric and catalytic sites of Sos, we recorded two-dimensional ^1^H,^13^C HMQC spectra of a sample of Sos^Cat^ (60 μm) that was uniformly ^13^C-labeled (34) at the Met (ϵ-) and Ile (δ_1_-)methyl positions (that we call for brevity [^13^C-methyl-Met,Ile]Sos^Cat^). The methyl resonances from 10 labeled Met residues occurring in Sos^Cat^ are well resolved (Fig. 4*A*), whereas the methyls from 39 Ile residues suffer from significant spectral overlap in the HMQC spectra (Fig. 4*B*). Therefore, we focused on methyl-Met signals. To obtain the methyl resonance assignments, we consecutively substituted Ala for Met, creating 10 mutant forms of Ras: M563A, M567A, M592A, M617A, M714A, M726A, M824A, M878A, M997A, and M1001A. The absence of methyl signal in the spectrum of the mutant allows the assignment for this methionine in the spectrum of the WT protein (Fig. 5, *A* and *B*). Through this comprehensive mutagenesis approach, eight of the 10 Met methyl signals were unambiguously assigned (Fig. 4*A*). The spectra of M567A and M997A yielded no clear changes relative to the WT spectrum, which, with one unassigned peak remaining, suggests that the methyl signals from these two residues overlap with each other and that both contribute to this remaining unassigned peak.

Mapping the position of Met residues onto the crystal structure of the Ras/Sos complex (10) and relating them to positions of allosteric and catalytic binding sites (Figs. 4*C* and 5, *C* and *D*), it is possible to separate signals into groups as originating from (and presumably primarily reporting on) allosteric (a) and catalytic (c) sites. A third group of Met residues is positioned in between the sites (a/c) and thus may report on structural changes in Sos in response to binding at either of these sites. For convenience, we will indicate the site-specific origin of these potential reporter signals with corresponding superscripts with M563^(a)^, M592^(a)^, M617^(a)^, and M726^(a)^ arising from the allosteric site of Sos and residues M824^(c)^, M878^(c)^, and M1001^(c)^ arising from the catalytic site. M714^(a)/(c)^ is positioned in between the allosteric and catalytic sites and therefore belongs to the third group. Subsequent titration experiments (see below) showed that in fact only three of these potential reporters, M726^(a)^, M824^(c)^, and M714^(a)/(c)^, display appreciable sensitivity to Ras binding at the allosteric and catalytic sites.

##### Monitoring NMR Signal Perturbations of Sos^Cat^ upon Addition of H-Ras^NF^

**FIGURE 6.**
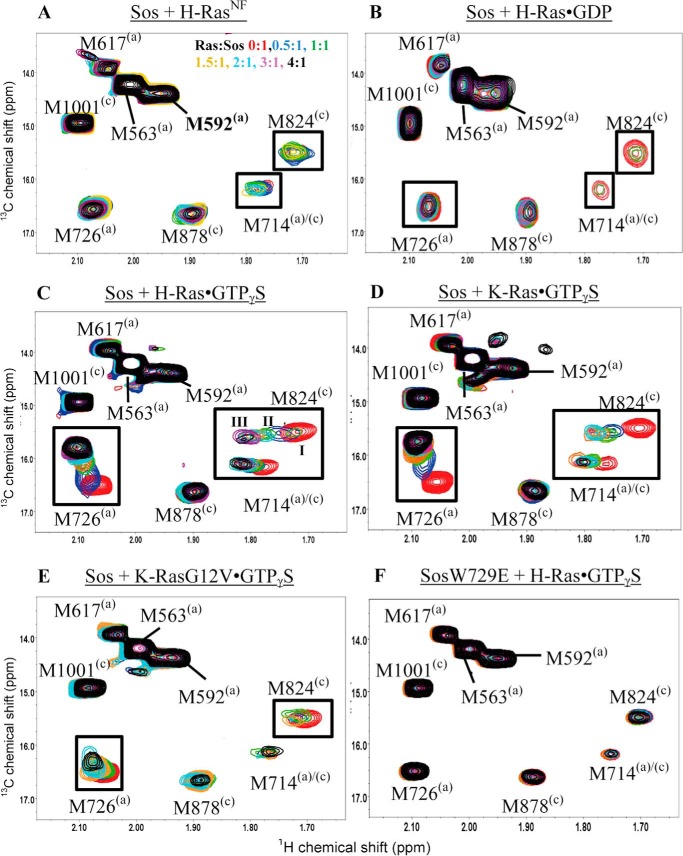
**Monitoring changes in the ^1^H,^13^C HMQC spectra of Sos^Cat^ upon the addition of Ras.** The Met methyl resonances of Sos^Cat^ (*A–E*) (*red*) and Sos^Cat^W729E (*F*) (*red*) were monitored upon the addition of Ras at a Ras:Sos stoichiometry of 0.5:1 (*blue*), 1:1 (*green*), 1.5:1 (*orange*), 2:1 (*cyan*), 3:1 (*purple*), and 4:1 (*black*) in the HMQC spectra. The titrated Ras form is H-Ras^NF^ (A), H-Ras·GDP (*B*), H-Ras·GTPγS (*C*), K-Ras·GTPγS (*D*), K-RasG12V·GTPγS (*E*), and H-Ras·GTPγS (*F*). Chemical shift perturbations and signal broadening are indicated as *black boxes* and *dashed boxes*, respectively. Characteristic states occurring for the M824^(c)^ signal upon addition of H-Ras·GTPγS are marked in *C* as *I*, *II*, and *III*.

To investigate the binding of Sos^Cat^ to nucleotide-free H-Ras, increasing amounts of unlabeled H-Ras^NF^ were added to a sample containing [^13^C-methyl-Met,Ile]Sos^Cat^ (Fig. 6*A*). The addition of H-Ras^NF^ at higher concentrations, *e.g.* above 2:1 Ras:Sos stoichiometry, showed severe broadening for the M824^(c)^ resonance (Fig. 6*A*), suggesting that H-Ras^NF^ binds at the catalytic site of Sos^Cat^. Furthermore, slight signal broadening was also observed for M714^(a)/(c)^. Residue M714^(a)/(c)^ is located near the core of Sos and is therefore possibly sensitive to the conformational and/or dynamic changes induced in Sos upon H-Ras^NF^ binding. Most importantly, residue M726^(a)^ (which turned out to be the most sensitive probe for binding at the allosteric site; see below) was largely unperturbed, suggesting that H-Ras^NF^ does not bind at the allosteric site. Our site-specific results therefore indicate that H-Ras^NF^ in solution can only bind to the catalytic site of Sos. This complements previous crystal studies of H-Ras and Sos (8, 10) showing that nucleotide-free H-Ras only ever bound at the catalytic site.

##### Monitoring Signal Perturbations of Sos^Cat^ upon H-Ras·GDP Binding

Binding of Ras·GDP to Sos promotes a basal level of nucleotide exchange activity that is observed in GDP → GDP exchange assays (11–13). Ras·GDP binding to the allosteric site is believed to induce weak nucleotide exchange activity at the catalytic site (11). Here, we explored directly to which of the sites H-Ras·GDP binds by adding unlabeled Ras·GDP to [^13^C-methyl-Met,Ile]Sos^Cat^. The methyl signals of Sos M714^(a)/(c)^ and M824^(c)^ are heavily broadened in the HMQC spectra of 2:1 and 4:1 H-Ras·GDP:Sos samples, whereas these signals are still visible in the spectrum of a 1:1 H-Ras·GDP:Sos sample (Fig. 6*B*). Signal broadening of M714^(a)/(c)^ in Sos indicates that H-Ras·GDP may cause some conformational or dynamic changes to Sos. Interestingly, M726^(a)^ was not significantly perturbed even at 4-fold excess of H-Ras·GDP, suggesting that Ras·GDP does not associate noticeably with the allosteric site of Sos^Cat^. Importantly, the broadening of M824^(c)^ signal confirms that H-Ras·GDP binds to the catalytic site (Fig. 6*B*), suggesting that binding to the catalytic site can occur in the absence of significant occupancy of the allosteric site. Overall, the binding is relatively weak in agreement with our other NMR (Fig. 3*B*) and fluorescence data (Table 1). Preferential weak binding of H-Ras·GDP to the catalytic site of Sos, but not to the allosteric site, suggests that H-Ras·GDP is not expected to play a significant role in activating Sos via allosteric interactions and that the basal, low level activity of Sos for GDP → GDP exchange in the absence of GTP may be due to inherent weak binding of Ras·GDP to the catalytic site only. The role of DH-PH and histone domains of Sos (11, 32) in further down-regulating its basal catalytic activity thus may be more subtle than just sterically blocking the allosteric site for Ras·GDP (as well as for Ras·GTP) binding.

##### Monitoring Signal Perturbations of Sos^Cat^ upon H-Ras·GTPγS Binding

**FIGURE 7.**
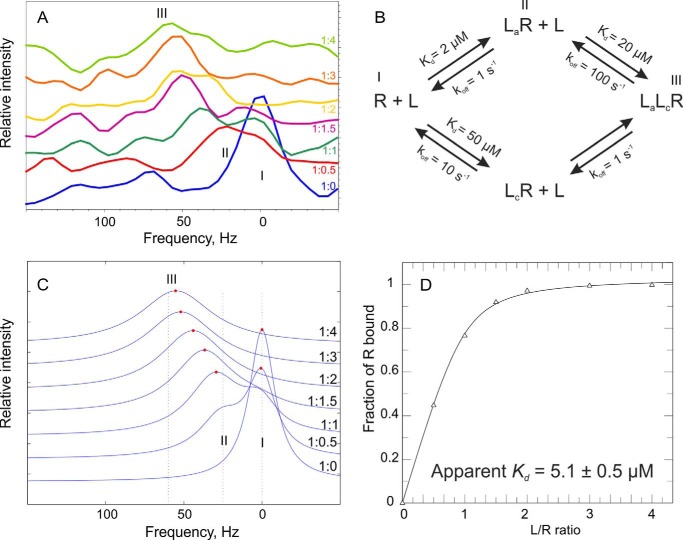
**Consequences of two-site binding and chemical exchange rates for NMR signal shifts and apparent binding constants.** *A*, horizontal slices through the M824^(c)^ cross-peak of Sos^Cat^ showing relative spectral changes in the ^1^H dimension upon addition of the specified equivalents of H-Ras·GTPγS. The peak positions for binding States I, II, and III are marked. *B*, thermodynamic cycle for two-site binding reaction between receptor (*R*) and ligand (*L*) with plausible values of *K_d_* and off-rates (*k*_off_) used for simulation with LineShapeKin software (35). *L_a_* and *L_c_* denote ligands bound at the allosteric and catalytic sites, respectively, and binding States I, II, and III are labeled accordingly. The simulated spectral traces (with [receptor] fixed at 60 μm) shown in *C* mimic qualitatively the behavior of experimental spectra presented in *A*. State I corresponds to the chemical shift of free Sos^Cat^, state II corresponds to H-Ras·GTPγS bound tightly (in slow exchange) at the allosteric site, and state III corresponds to a second H-Ras·GTPγS molecule binding weakly (in fast exchange) at the catalytic site. The simulated dependence of the fraction of receptor bound *versus* the [ligand]:[receptor] (*L/R*) ratio, however, can be easily fitted into a one-site binding isotherm (*D*), which yields an apparent “macroscopic” *K_d_* value of ∼5 μm, a value close to that measured for Sos^Cat^/H-Ras·GTPγS binding using fluorescence (see Table 1).

Ras·GTP binding to the allosteric site of Sos is expected to lead to Sos activation (10), whereas the ability of Ras·GTP to bind at the catalytic site of activated Sos has always been implied in the GTP → GTP exchange assays (11, 13). However, to our knowledge, the latter assumption has not been tested before. Here, binding between Ras·GTP and Sos was monitored in a site-specific manner via the HMQC spectra of [^13^C-methyl-Met,Ile]Sos^Cat^ upon addition of unlabeled H-Ras·GTPγS. The signals from M726^(a)^, M824^(c)^, and to a smaller extent M714^(a)/(c)^ shifted in the spectra (Fig. 6*C*). Residue M726 from the allosteric site exhibited the largest chemical shift change. In the crystal structure of the complex, the side chain of Sos M726^(a)^ is located close to residues I36 and E37 from the Switch I region and Y64 from the Switch II region of H-Ras (Fig. 5*D*). Residues M714^(a)/(c)^ and M824^(c)^ also showed significant signal perturbations and complex signal movement occurring simultaneously with perturbations at the allosteric site. Interestingly, close inspection of the M824^(c)^ peak changes during the titration (Figs. 6*C* and 7*A*) reveals that at 1:1 ratio there are two M824^(c)^ signals observed in slow exchange between State I (free form) and State II (remodeled form); with further addition of H-Ras·GTPγS, State I disappears, and the signal is gradually shifted from State II toward State III (fully bound) where it remains stable even at higher protein ratios. This peak movement suggests the existence of complex dynamic and structural rearrangements at the catalytic site in response to initial binding at the allosteric site. Although overall the initial perturbation to M824^(c)^ appears to be dominated by conformational changes in response to binding at the allosteric site, further gradual signal shifts reveal that there may be secondary, weaker binding occurring for Ras·GTP at the catalytic site that is in the fast exchange regime on the chemical shift time scale. Our fluorescence experiments where binding to the allosteric site was partially saturated with the addition of H-RasY64A·GTPγS (Table 1) concurred with only weak binding of H-Ras·GTPγS at the catalytic site. In addition to the shift changes observed on the [^13^C]methyl of Met, several of the non-assigned [^13^C]methyl-Ile resonances were also significantly perturbed in the presence of H-Ras·GTPγS (data not shown). The complex peak movements observed here and the presence of both slow and fast chemical exchange regimes may limit the reliability of signal shifts as a measure of the fraction of protein bound to the ligand (35), which would complicate estimates of *K_d_* values from these signal shifts. Indeed, we could not obtain a good fit for the dependence of signal shifts on concentration to a single site binding model either for allosteric or for catalytic site signals. Moreover, because of the low protein concentrations used here and the large size of Sos^Cat^ itself and its complexes with H-Ras, the signal-to-noise ratio was too poor to quantify signal intensities of methyl signals throughout the titration (Fig. 7*A*), which otherwise could have been used to characterize the exchange regimes further. However, we ran simulations (Fig. 7, *B–D*) that show that the signal shift behavior for M824^(c)^ signal can be reasonably recreated for a two-binding site model with slow and fast exchange regimes. More accurate measurements at much higher protein concentrations and with more experimental points would be required to extract qualitative information about the exchange rates in this system. Taken together, the observations by NMR and fluorescence suggest that the overall binding of H-Ras·GTPγS to Sos^Cat^ is dominated by binding at the allosteric site, and its binding to the catalytic site is much weaker than to the allosteric site.

##### Monitoring Signal Perturbations of Sos^Cat^ upon K-Ras·GTPγS Binding

To explore whether K-Ras binding to Sos differs from that of H-Ras, we titrated unlabeled K-Ras·GTPγS into [^13^C]methyl-Ile,Met-labeled Sos^Cat^. The signals from M714^(a)/(c)^, M726^(a)^, and M824^(c)^ showed the most significant changes in the spectrum (Fig. 6*D*). In addition to the shift changes observed on the [^13^C]methyl of Met, several of the [^13^C]methyl-Ile residues were also significantly perturbed in the presence of K-Ras·GTPγS (data not shown). The pattern of perturbation is comparable with that caused by H-Ras·GTPγS. However, the slow conformational exchange observed at the catalytic site in response to occupation of the allosteric site by H-Ras was not detected for K-Ras binding, suggesting that there may be subtle differences in how these Ras isoforms affect the dynamics of Sos and/or slight differences in binding site affinities. The apparent microscopic *K_d_* value (∼2 μm) estimated from the M726^(a)^ signal shift, which represents K-Ras·GTPγS binding at the allosteric site, is much lower than the apparent macroscopic *K_d_* measured by fluorescence for K-Ras binding to Sos^Cat^ with the assumption of single site binding model (Table 1), again suggesting that binding at the allosteric site dominates the binding at low ligand concentrations. Because the chemical shift changes follow the same pattern upon H-Ras·GTPγS or K-Ras·GTPγS addition to Sos^Cat^, it is likely that Sos^Cat^ adopts a similar conformation albeit accompanied by subtly different dynamic perturbations.

##### Monitoring Signal Perturbations in Sos^Cat^ upon K-RasG12V·GTPγS Binding

To examine whether the binding of the K-RasG12V mutant to Sos^Cat^ is different in any way from its wild-type variant, we recorded HMQC spectra of [^13^C-methyl-Met,Ile]Sos^Cat^ upon the addition of unlabeled K-RasG12V·GTPγS. The M824^(c)^ signal in the catalytic site of Sos^Cat^ undergoes only a minor shift but does decrease in intensity and broaden when above equimolar concentrations of K-Ras are added (Fig. 6*E*). The signal perturbations for M726^(a)^ in the allosteric site are less pronounced than those observed with WT K-Ras, indicating that binding to the allosteric site of Sos is weaker for the Ras mutant (Fig. 6*E*). These findings are supported by the binding measurements obtained from our fluorescence studies, which revealed that the affinity for K-RasG12V·GTPγS binding to Sos (*K_d_* = 31 μm) was weaker than that of WT K-Ras (*K_d_* = 10 μm). This suggests that the G12V mutant of K-Ras, which is one of the most frequently observed somatic Ras mutations in cancers, is compromised in its ability to bind Sos at the allosteric site and activate it compared with the wild type.

##### Monitoring Signal Perturbations in W729E Mutant Sos^Cat^ upon K-RasG12V·GTPγS Binding

To characterize the binding of Ras·GTP at the catalytic site in the absence of allosteric activation of Sos, the allosteric site binding can be impaired through mutation of W729 of Sos^Cat^ to Glu (W729E) (11). Upon addition of unlabeled H-Ras·GTPγS to [^13^C-methyl-Met,Ile]Sos^Cat^W729E, no perturbations were observed for any of the characteristic signals, including M726^(a)^, M714^(a)/(c)^, and M824^(c)^, even when overtitrated (Fig. 6*F*), suggesting that there is no detectable binding to either of the binding sites. The fluorescence measurements for this interaction revealed a macroscopic *K_d_* of 28 μm (Table 1), which may have been dominated by residual non-optimal binding to the sites away from the reporter residues used in the NMR. This value is comparable with the *K_d_* of 32 μm obtained from fluorescence measurements for the interaction between Sos^Cat^W729E and H-RasY64A·GTPγS, a combination of mutants that is supposed to block interactions at both allosteric and catalytic sites. The NMR data overall confirm that the W729E mutation inhibits interaction with Ras·GTP at the allosteric site, preventing Sos activation and further binding of Ras·GTP at the catalytic site.

## Discussion

In our fluorescence-based studies, we monitored the intrinsic fluorescence of natively occurring Trp residues in Sos^Cat^ and Sos^HD-DH-PH-Cat^ upon addition of Ras, which is devoid of fluorophores, to avoid any possibility of perturbing protein structure or binding activity as may be otherwise expected from using extrinsic dyes (16). As an orthogonal and complementary approach, we also used NMR to report on the details of Ras and Sos interactions from the viewpoints of both interacting partners in a site-specific manner. Using [^13^C]methyl NMR probes for monitoring changes in structure and dynamics is innately non-perturbative and therefore highly robust (34, 36–38). We identified three useful Sos^Cat^ methyl groups in M726^(a)^, M824^(c)^, and M714^(a)/(c)^, which can be conveniently detected in well resolved ^1^H,^13^C HMQC spectra and which report from allosteric and catalytic sites as well as from the interface between them. These signals are sensitive to all aspects (structural as well as dynamic) of Ras-Sos interaction and can be used for exploring mechanistic aspects of Sos function in various complexes. This strategy, applied consistently to a variety of combinations of different Ras isoforms (K-Ras, H-Ras, and their selected mutants) in different nucleotide-loaded states (nucleotide-free, GDP-loaded, or loaded with GTP analogs) and different Sos constructs (Sos^HD-DH-PH-Cat^, Sos^Cat^, and its mutants), allowed us to obtain for the first time site-specific information on the localized binding events at the allosteric and catalytic sites of Sos.

**FIGURE 8.**
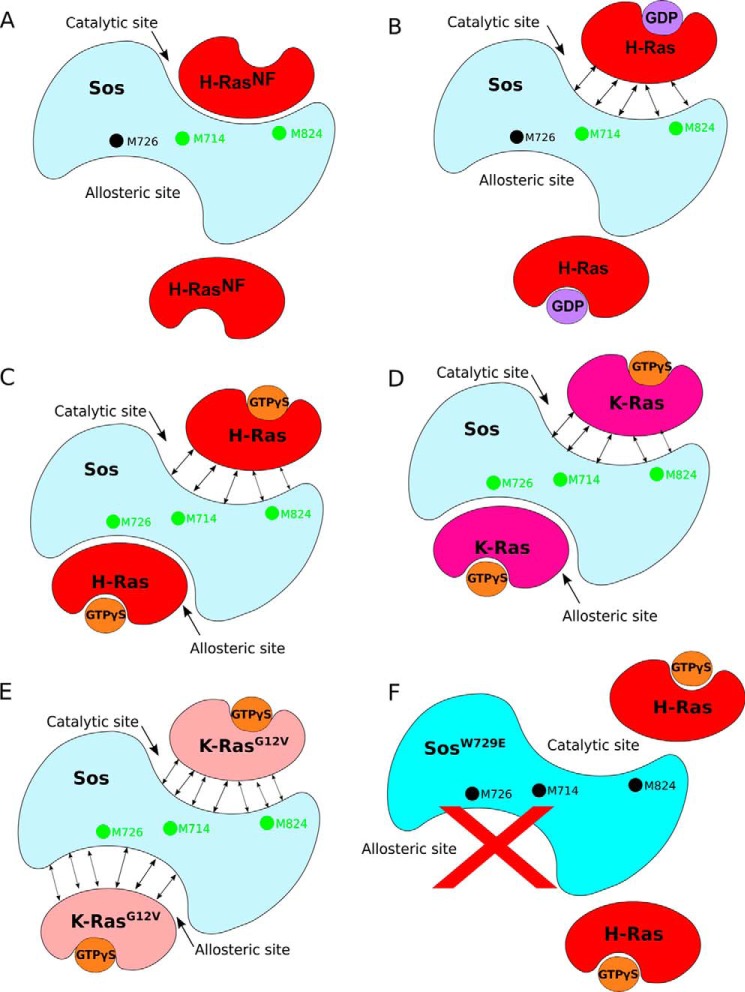
**Proposed modes of Ras binding to Sos from our NMR assay.** *A*, H-Ras^NF^ binds at the catalytic site of Sos and perturbs residues M824^(c)^ and M714^(a)/(c)^ only (*green dots*). *B*, H-Ras·GDP binding to the catalytic site only slightly perturbs Sos residues M714^(a)/(c)^ and M824^(c)^. Unperturbed signal from Sos M726^(a)^ is indicated as a *black dot. Double-ended arrows* indicate weak interactions. *C*, H-Ras·GTPγS binds at the allosteric site of Sos as confirmed by the perturbation of residue M726^(a)^. The binding of H-Ras·GTPγS at the allosteric site induces a conformational change to Sos, detected by the perturbation of residues M714^(a)/(c)^ and M824^(c)^, that allows a second H-Ras·GTPγS to bind weakly to the catalytic site. *D*, the mechanism for K-Ras·GTPγS is similar to that for H-Ras·GTPγS. *E*, K-RasG12V·GTPγS binds to allosteric and catalytic sites only weakly. *F*, no Ras binding was observed at both the allosteric and catalytic sites of SosW729E mutant, which blocks Ras binding to the allosteric site.

Taken together, our results are presented schematically in Fig. 8. The nucleotide-free form of H-Ras (which is not a species that is significantly populated in isolation *in vivo* but may usefully represent a transition state) binds at the catalytic site only, affecting only the specific reporter signal M824^(c)^ (Fig. 8*A*). Note that in this interaction Sos is not activated due to the lack of allosteric site binding. The catalytic site binding also leads to significant signal perturbations in H-Ras^NF^ itself. These results agree with crystallography studies where H-Ras^NF^ has only ever been found at the catalytic site of Sos (8, 10). Binding of GDP-loaded H-Ras to the catalytic site of non-activated Sos^Cat^ appears to be much weaker than that of Ras^NF^ (Fig. 8*B* and Table 1) as judged by signal perturbations on Sos^Cat^ and Ras. Importantly, the NMR data suggest that H-Ras·GDP cannot bind at the allosteric site strongly enough to achieve significant activation of Sos; this is in agreement with previous observations (11) but also suggests that the level of Sos activation by Ras·GDP in the recent single molecule studies (13) may have been overestimated. Weak transient binding of Ras·GDP at the catalytic site (even without activation), however, would explain the modest homonucleotide GDP* → GDP exchange routinely observed in nucleotide release assays (10, 11, 13).

As expected, H-Ras·GTPγS was found to interact strongly with the allosteric site of Sos^Cat^ (Fig. 8*C*), inducing strong signal shifts. Unexpectedly, binding of H-Ras·GTPγS at the catalytic site of fully activated Sos^Cat^ was only weak and transient. This is in agreement with the result from saturating the allosteric site with H-RasY64A·GTPγS, a mutant that only binds at the allosteric site (11, 12), and measuring H-Ras·GTPγS affinity at the catalytic site using fluorescence. The estimated lower limit of *K_d_* (≥21 μm) for Ras·GTPγS binding to the catalytic site of activated Sos is significantly higher than the *K_d_* of H-Ras·GDP at the same site (1.9 μm) (11). Having a high affinity for Ras·GDP, which is the natural substrate for the catalytic reaction, and low affinity for Ras·GTP, which is the natural reaction product, provides a previously underappreciated preferred direction for the nucleotide exchange reaction, GDP → GTP. In a situation when both GDP and GTP are present in solution (*e.g.* in cytosol), activated Sos would preferentially recognize Ras·GDP molecules with its catalytic site: once the exchange for GTP is complete, Ras·GTP would be released due to its lower affinity. To our knowledge, this is the first suggestion that the native nucleotide exchange mediated by Sos can have a preferred directionality and is not just a passive reloading of Ras molecules with a nucleotide according to the GTP:GDP ratio present in solution (3).

The NMR mapping experiments performed with K-Ras revealed results similar to those with H-Ras (Fig. 8*D*), although for K-Ras, the slow exchange at the catalytic site was not detected, and overall binding was weaker (Table 1). The binding of H-Ras and K-Ras to Sos may therefore be subtly different in terms of dynamics and affinity despite structural similarities and very similar nucleotide exchange and hydrolysis properties (39). Interestingly, the mutation G12V of K-Ras showed substantially reduced binding to Sos (Table 1). Unlike the WT, the mutant lacks the ability to induce strong signal perturbations at the allosteric site of Sos and only weakly binds at allosteric and catalytic sites (Fig. 8*E*). Therefore, the G12V mutant is likely to be defective in its ability to bind Sos and activate it via allosteric site binding. Finally, the W729E mutant of Sos^Cat^, which is known to block the interaction with Ras·GTP at the allosteric site (11), did not show any signal perturbations either at the allosteric or catalytic sites upon addition of H-Ras·GTPγS (Fig. 8*F*), which confirms that in solution binding of Ras·GTP at the allosteric site is absolutely required to induce binding of Ras·GTP at the catalytic site. Similar results were also obtained for the Sos^HD-DH-PH-Cat^ construct (Table 1), which sterically occludes the allosteric site.

**FIGURE 9.**
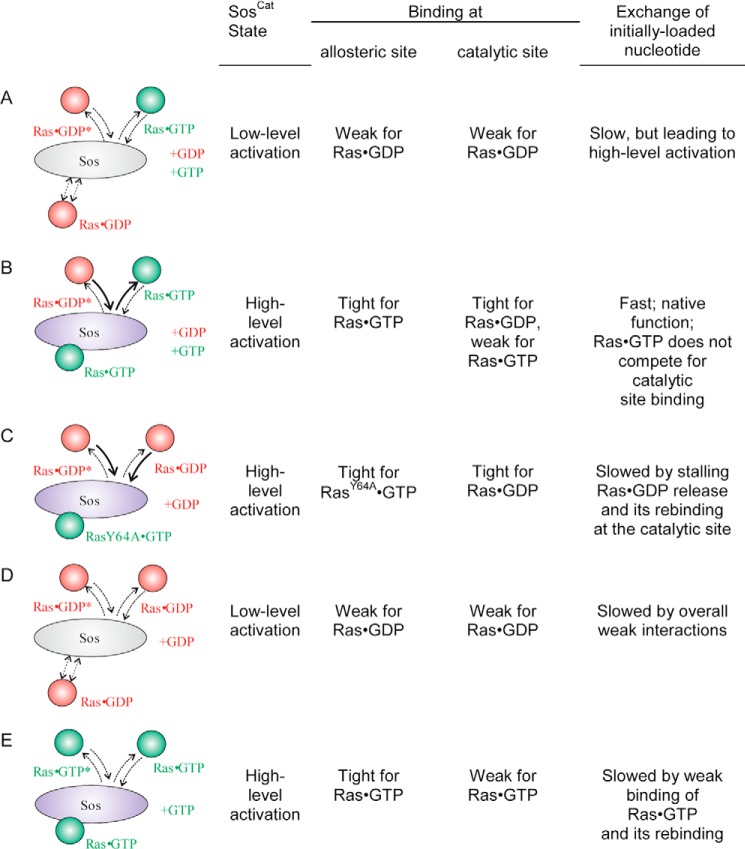
**Common scenarios for nucleotide exchange regimes in Ras/Sos system.** The allosteric site is schematically shown at the *bottom* of Sos and catalytic site is schematically shown at the *top*. For each scenario, the state of the system and qualitative description of binding are shown on the *right*, including the expected exchange rate of the nucleotide that was initially bound to incoming Ras. Scenario *A* corresponds to initial low level activation of Sos when no Ras·GTP is present yet with GDP → GTP exchange driven by the excess of GTP over GDP in the cytosol. Once enough Ras·GTP molecules are produced, they lead to positive feedback activation of Sos (scenario *B*) where the affinity at the catalytic site is high for Ras·GDP and low for Ras·GTP, leading to efficient, native turnover and expected maximum GDP → GTP exchange rate. Both nucleotides (+*GDP* and +*GTP*) are present. In scenarios *C–E*, typical for assays where only one type of nucleotide is present in excess, a release of labeled nucleotide (marked with *) initially bound to the catalytic site is expected to be slowed down (relative to the native rate for scenario *B*) due to either stalling of Sos recycling (scenario *C*) or the presence of weak binding bottleneck steps at the allosteric (*D*) or catalytic site (*E*).

The low affinity binding of Ras·GTP to the catalytic site of Sos highlighted in this study may have implications for how nucleotide exchange assays mediated by Sos are run and interpreted. The main scenarios of nucleotide exchange, together with the consequences of binding at each individual site, are presented in Fig. 9. For simplicity, additional down-regulation of Ras by N-terminal domains of Sos occluding the allosteric site (14) is omitted. Whereas scenario A represents early events in Sos activation and promoting GDP → GTP exchange on Ras, scenario B is expected to represent the native, functional GDP → GTP exchange, which should be the fastest and the most efficient. It relies on strong binding of Ras·GTP to the allosteric site, activating Sos; strong binding of Ras·GDP (the substrate) to the catalytic site; and fast release of Ras·GTP (the product) from the catalytic site once the exchange there is complete. A number of studies conducted some assays in these conditions (17, 32, 40–42). However, some nucleotide release assays quantified homonucleotide exchange as a measure of Sos activity and followed scenarios C–E with each of those suffering from one or several bottleneck interactions (see Fig. 9) expected to slow down the apparent rate of nucleotide release or exchange. In these cases, the activity of Sos as measured by homonucleotide GDP → GDP or GTP → GTP exchange rates (10, 11, 13, 40, 43) may be underestimated. Recently, very high heteronucleotide GDP → GTP exchange rates of 0.28 s^−1^ were reported in bulk assays that used non-modified nucleotides and biosensor detection (41): the S shape of time courses (see Fig. 4A in Ref. 41) is consistent with the initial low level activation quickly followed by the full activation as is expected from combined scenarios A and B. Membrane tethering of Ras was shown to increase the rate of nucleotide exchange ∼500-fold compared with the bulk assays (32), but interestingly, the experiments done by these authors also reveal that the rate of heteronucleotide GDP → GTP exchange reaction is a further ∼10 times faster than homonucleotide GDP → GDP in the identical conditions, reaching a rate of ∼5 s^−1^ (see supplemental Fig. 2 in Ref. 32). Even when Sos was activated by the presence of RasY64A loaded with a stable GTP analog, the homonucleotide exchange reaction rate was much lower (32). The marked increase in the rates for heteronucleotide exchange was also consistently observed in the presence of the N-terminal segment of Sos that down-regulates its activity (32). In another bulk assay study (40), the rates of Sos^Cat^-catalyzed reactions for mantGDP → GTP and mantGTP → GTP exchanges were measured. In their study, addition of equimolar Sos^Cat^ to WT Ras increased the rates of exchange (relative to the intrinsic rates) by factors of 112 and 21, respectively, showing that fully activated Sos^Cat^ enhances the rate of the heteronucleotide exchange reaction about 5 times faster than the rate of the homonucleotide GTP → GTP exchange reaction (see Table 2 of Ref. 40). This consistent increase of observed rates for native heteronucleotide exchange in various previous experiments can now be explained by the constructive combination of the stronger (∼10-fold) binding and activation of Sos by Ras·GTP (compared with Ras·GDP) at the allosteric site (11) and the at least 10-fold greater binding affinity of the catalytic site for Ras·GDP (compared with Ras·GTP), which was revealed in this study. Thus, our results and a re-evaluation of previously published data suggest that an adequate representation of the nucleotide exchange catalyzed by Sos in assays may need to preferably measure the GDP → GTP exchange reaction and be conducted in the presence of both Ras·GTP (to activate Sos) and Ras·GDP (as the native substrate for the catalytic conversion to Ras·GTP) and in the presence of an excess of GTP or its analog.

In conclusion, from our NMR Ras-Sos interaction assay developed and presented here and experiments on different Ras forms, we were able to disentangle nucleotide-dependent Ras binding at the allosteric and catalytic sites of Sos. To achieve a fully functional heterotrimeric complex with Sos, *e.g.* to study its functioning mechanism, both GDP- and GTP-loaded Ras molecules should be present. Our solution studies fully support previously proposed mechanism for positive feedback activation of Sos (10) but also suggest that the extent of such activation may have been previously underestimated when the homonucleotide exchange rate was measured. The NMR approach described here opens new avenues through which to investigate this complex process directly in more detail in the future. Similarly, further NMR experiments can shed light on the molecular level structural and dynamic detail of the processes involved in self-activating Sos by Ras (13). The site-specific interaction assay presented here may also aid the development and screening of future drugs designed against Ras-Sos interactions at particular sites of Sos (44).

## Author Contributions

K. J. E., A. P. G., and A. L. B. conceived and designed the project. U. V., N. V., L. F., and R. B. performed experiments and contributed materials. U. V., A. P. G., and K. J. E. wrote the manuscript. All authors contributed to the final manuscript.

## Footnotes

5 The abbreviations used are:

Sos: Son of Sevenless
HMQC: heteronuclear multiple quantum coherence
Ras^NF^: nucleotide-free form of Ras
TROSY: transverse optimized spectroscopy
DH: Dbl homology
PH: pleckstrin homology
GTPγS: guanosine 5′-(γ-thio)triphosphate
GppNp: guanosine 5′-[(β,γ)-imino]triphosphate
GppCp: guanosine 5′-[(β,γ)-methyleno]triphosphate
mantGTP: 2′/3′-*O*-(*N*-methylanthraniloyl)-guanosine 5′-triphosphate
mantGDP: 2′/3′-*O*-(*N*-methylanthraniloyl)-guanosine 5′-diphosphate
(a): allosteric site
(c): catalytic site.
